# A new approach to dual-color two-photon microscopy with fluorescent proteins

**DOI:** 10.1186/1472-6750-10-6

**Published:** 2010-02-02

**Authors:** Shane E Tillo, Thomas E Hughes, Nikolay S Makarov, Aleks Rebane, Mikhail Drobizhev

**Affiliations:** 1 Department of Cell Biology and Neuroscience, Montana State University, Bozeman, MT 59717, USA; 2 Department of Physics, Montana State University, Bozeman, MT 59717, USA; 3 Department of Physics, Montana State University, Bozeman, MT 59717, USA and National Institute of Chemical Physics and Biophysics, Tallinn, EE 12618, Estonia

## Abstract

**Background:**

Two-photon dual-color imaging of tissues and cells labeled with fluorescent proteins (FPs) is challenging because most two-photon microscopes only provide one laser excitation wavelength at a time. At present, methods for two-photon dual-color imaging are limited due to the requirement of large differences in Stokes shifts between the FPs used and their low two-photon absorption (2PA) efficiency.

**Results:**

Here we present a new method of dual-color two-photon microscopy that uses the simultaneous excitation of the lowest-energy electronic transition of a blue fluorescent protein and a higher-energy electronic transition of a red fluorescent protein.

**Conclusion:**

Our method does not require large differences in Stokes shifts and can be extended to a variety of FP pairs with larger 2PA efficiency and more optimal imaging properties.

## Background

Double labeling is a powerful tool in microscopy, and there are many pairs of FPs that can be used for dual labeling in wide field and confocal microscopy. However, most two-photon microscopes are fitted with only one expensive, femtosecond laser, making dual-imaging with fluorescent proteins (FPs) difficult. The difficulty stems from the fact that a single excitation wavelength rarely produces well-separated fluorescence emission spectra. One solution is to use spectral unmixing of overlapping emission profiles. A drawback of this method is that it requires multiple detectors, which results in a loss of speed and sensitivity. A more traditional approach has been to find a pair of FPs with similar absorption wavelengths and large differences in their Stokes shifts (see methods: photophysical terms) such that a single excitation wavelength can produce well-separated emission spectra. Recently, Kawano et al. have proposed the use of the EGFP/mKeima combination at excitation wavelengths (810-1000 nm) where the lowest-energy, or long wavelength transitions of their chromophores overlap [1]. The discrimination of fluorescence signals is possible thanks to the extremely large (~180 nm) Stokes shift of mKeima [2]. The applicability of this approach is limited because large differences in Stokes shifts are not common among FP pairs, and mKeima and EGFP have relatively low two-photon absorption efficiency, or cross sections, at the optimal wavelength of excitation (900 nm) [1,3,4].

Recently, we performed a detailed characterization of the two-photon absorption (2PA) properties of a series of orange and red FPs over a wide range of wavelengths (650 -1300 nm) [3,5]. The spectra reveal optimal wavelengths for excitation, new excitable two-photon transitions, and the best absorbing and brightest FPs in the series for use in two-photon laser scanning microscopy (TPLSM) [6]. One of the proteins, tagRFP [7], has a high two-photon cross section in the range of 700-780 nm [3]. The two-photon absorption at this range of wavelengths is due to a higher-energy, or short wavelength transition(s) of the tagRFP chromophore and is within the range of the mode-locked Ti:sapphire lasers commonly found on commercially available two-photon microscopes.

We reasoned that if we could identify a FP with a strong lowest-energy, short wavelength transition, in the same 700 - 780 nm region, it would provide an ideal partner with tagRFP for two-photon dual-color imaging. In search of this partner FP we have collected 2PA spectra for a series of recently developed blue, teal, and green FPs [8-11]. These proteins are photostable, pH insensitive, have relatively high quantum yields and extinction coefficients, and work well in fusion tags (note that mTFP1.0b is mTFP1.0 R149K/V161I/F177K and ECFP contains the N164H mutation).

## Results and Discussion

**Figure 1:**
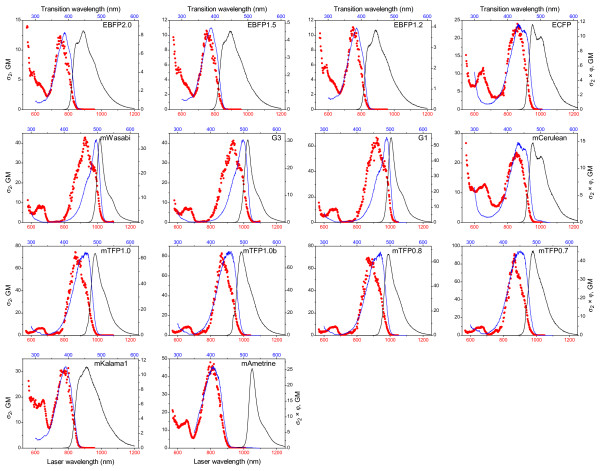
**Two-photon cross section and brightness of blue/teal/green FP series**. 2PA spectra (red circles), one-photon fluorescence excitation spectra (blue line), and fluorescence emission spectra (black line) of the FPs studied. The left-ordinate axis represents the two-photon cross section values (GM), and the right-ordinate axis represents the two-photon brightness, σ_2_φ, (GM). The bottom x-axis represents the laser wavelength used for excitation and the top x-axis represents the transition wavelength. The excitation and fluorescence emission intensities are shown in arbitrary units.

Figure 1 shows the 2PA spectra of the blue/teal/green FPs. The spectra of the classic cyan varieties, ECFP [12] and mCerulean [13], are included for comparison. The 2PA spectra of all the FPs are similar in regard that they consist of two distinct absorption bands. The longer wavelength band is attributed to the lowest-energy electronic (S_0_→S_1_) transition and the short wavelength band results from transitions to a higher-energy electronic state(s) (S_0_→S_n_). Partially resolved vibronic transitions give the longer wavelength band its complex structure, and with the exception of mAmetrine whose 1PA and 2PA maxima coincide, the dominant 2PA peak is blue-shifted compared to the 1PA spectrum due to a vibronic redistribution of intensity [3].

The FPs studied have the advantage that the longer wavelength 2PA band of their chromophores lies within the tuning range of commercial Ti:sapphire lasers commonly used in TPLSM. All of the FPs exhibit relatively high 2PA efficiency (cross section, σ_2_). Inspection of Figure 1 and Table 1 reveals that the FPs in the EBFP series (BFP Y66H chromophore) have very similar cross section values of the order of 10 GM at ~750 nm. Because of its high quantum yield and photostability [8], EBFP2 should be well-suited for TPLSM. The proteins in the mTFP series, G2, G3, and mWasabi, all of which contain the EGFP phenolate chromophore, have significant differences in their cross section values and optimal wavelengths of excitation. Out of this series, mTFP1 has the highest cross section, σ_2 _~75 GM, and G3 the lowest, σ_2 _~40 GM. mTFP0.7 absorbs at the shortest wavelength, 865 nm, and G3 at the longest wavelength at 935 nm. For imaging applications mTFP1 and mWasabi may be preferred due to their high quantum yields and photostability [9,11]. Two FPs, mKalama1 and mAmetrine, contain the GFP phenol chromophore. mKalama1 has a cross section of ~30 GM at 770 nm and absorbs fairly well at longer wavelengths. mAmetrine absorbs optimally at 810 nm and has a cross section of 45 GM. For comparison, mCerulean and ECFP (GFP Y66W chromophore), whose 2PA peaks also lie within the range of common Ti:sapphire lasers, have cross section values of ~25 GM at 855 nm.

**Table 1:** Summary of the optimum two-photon excitation wavelengths and corresponding peak cross section and peak brightness values (per chromophore) of the FPs studied.

Protein	λ_opt _(nm)	σ_2 _(λ_opt _)GM	σ_2_ϕ (λ_opt _)GM
mTFP1.0	867	75	65

mTFP1.0b	871	75	66

mTFP0.8	885	65	59

mTFP0.7	865	84	40

G1	918	63	52

G3	937	40	29

mWasabi	927	43	35

mKalama1	770	30	11

mAmetrine	809	45	26

EBFP2	750	12	7.7

EBFP1.5	750	10	4.3

EBFP1.2	750	11	4.0

ECFP(N164H)	857	23	11

mCerulean	858	23	13

Unlike many of the orange and red FPs [3], the higher-energy electronic (S_0_→S_n_) transitions of the proteins studied here are weaker than the lowest-energy electronic (S_0_→S_1_) transitions, and only the EBFP series, ECFP, and mCerulean demonstrate moderate resonant enhancement of the blue side of the S_0_→S_n _band. These results agree with quantum chemical calculations of the 2PA transitions present in these chromophores [14]. Note that the data shown here were collected only at wavelengths where the fluorescence signal showed a quadratic dependence on laser power, indicating that only two-photon absorption contributed to the signal.

**Figure 2:**
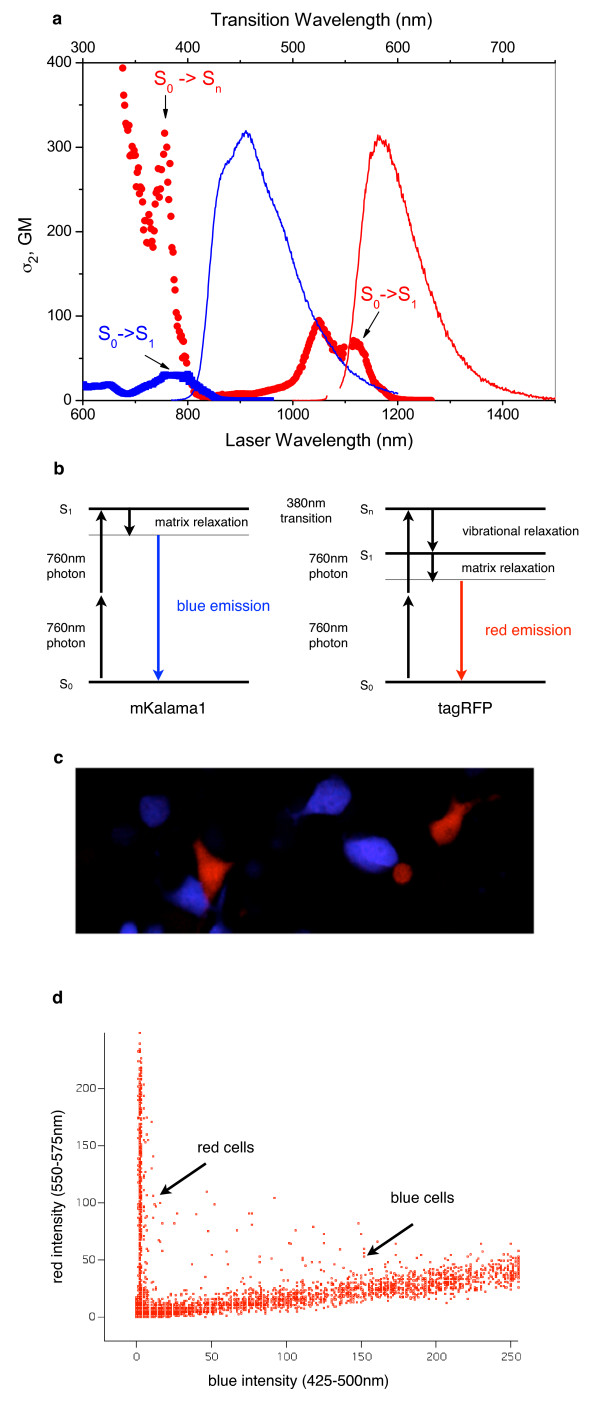
**TagRFP and mKalama1 are an optimal pair for dual-color imaging**. **(a) **2PA spectra of tagRFP (red circles) and mKalama1 (blue squares) and the fluorescence emission spectra of tagRFP (red line) and mKalama1(blue line). The y-axis represents the cross section (GM). The top x-axis represents the transition wavelength and the bottom x-axis represents the laser wavelength used for excitation. The different transitions of each FP chromophore are marked with arrows. Fluorescence emission spectra are shown in arbitrary units. **(b) **Jablonski diagram showing the electronic transitions that tagRFP and mKalama1 experience upon excitation with 760 nm light. **(c) **To test this approach we mixed HEK293 cells expressing either tagRFP or mKalama1 and imaged them with 780 nm excitation. (d) Scatter plot graphing red vs. blue pixel intensity of the image found in **(a)**. With the settings used, none of the red signal is picked up by the blue detector. However, because of the broad red-shoulder in the mKalama1 emission spectrum (Figure 2) a small amount of the signal in the red detector is due to blue emission, as indicated by the slight linear slope found in the blue cells.

These spectra reveal that mKalama1 is particularly well suited as a partner with tagRFP in two-photon dual-color imaging. Figure 2(a) shows the 2PA spectra of tagRFP (red circles) and mKalama1 (blue squares) grouped together with their corresponding fluorescence emission spectra (red and blue lines, respectively). For reference, the different electronic transitions present in the FP chromophores are marked with arrows. The lowest-energy electronic (S_0_→S_1_) transition of mKalama1 overlaps significantly with a higher-energy electronic (S_0_→S_n_) transition(s) of tagRFP. At an excitation wavelength of 760 nm, tagRFP possesses a cross section of ~300 GM and two photon brightness of ~130 GM, and mKalama1 a cross section of ~30 GM and two-photon brightness of 11 GM. Note also that the fluorescence emission spectra are well separated with a 120 nm spread between the peaks, despite neither FP having a large Stokes shift (Table 2). Figure 2(b) shows a Jablonski diagram depicting the electronic transitions in the mKalama1 and tagRFP chromophores upon excitation with 760 nm light. Note that Fluorescence only occurs from the S_1 _state [15] and the emitted photons differ significantly in energy. When the excitation laser is tuned to 780 nm, it should excite both FPs, and band pass filters should make it possible to detect the two different emissions simultaneously. To test this approach, we transiently expressed tagRFP and mKalama1 in different HEK293 cells and mixed them. Figure 2(c) is an image of that mixture excited at 780 nm with 425 - 500 nm and 550 - 575 nm emission filters. To quantify the ability to detect each signal independently, we plotted each pixel in the image (Figure 2d). Because of the red shoulder in the mKalama1 emission spectrum there was some cross talk such that very bright blue signals were detected slightly by the red detector.

**Table 2:** Summary of the absorption and fluorescence emission data relevant to the evaluation of the extinction coefficient ε_max _using the Strickler-Berg equation.

Protein	λMax emission (nm)	τ (ns)	Quantum Yield (ϕ)	τ_R_	λMax absorption (nm)	ε_MAX_ **10**^**3**^**M**^-**1**^**cm**^-**1**^
mTFP1.0	494	3.2	0.87 ± 0.09	3.7	463	49.6

mTFP1.0b	492	3.14	0.88 ± 0.09	3.5	462	48.1

mTFP0.8	494	3.63	0.91 ± 0.09	3.8	471	46.5

mTFP0.7	489	1.82	0.48 ± 0.05	3.8	453	43.4

G1	504	3.29	0.83 ± 0.12	4.0	490	71.2

G3	512	2.99	0.73 ± 0.07	4.1	498	68.0

mWasabi	508	3.4	0.81 ± 0.08	4.2	498	73.2

mKalama1	455	1.84	0.35 ± 0.07	4.7	392	25.7

mAmetrine	525	3.93	0.57 ± 0.13	7.5	407	28.7

EBFP2	446	3.65	0.64 ± 0.13	5.3	390	24.0

EBFP1.5	448	2.4	0.43 ± 0.09	6.2	389	23.5

EBFP1.2	443	2.16	0.36 ± 0.07	5.7	386	21.8

mCerulean	477	3.42	0.55 ± 0.05	6.2	431	25.1

ECFP(N164H)	476	3.36	0.49 ± 0.05	6.9	433	22.7

## Conclusions

**Figure 3:**
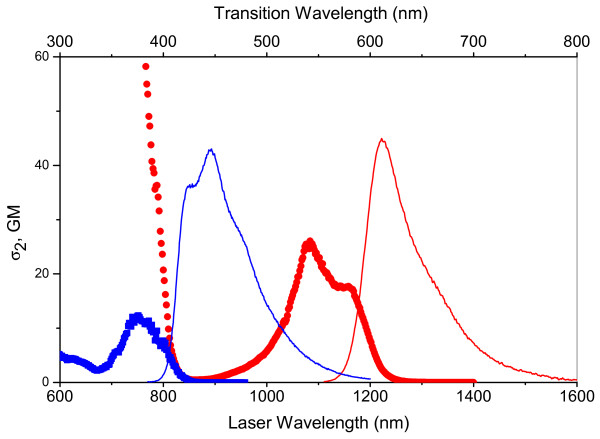
**mCherry and EBFP2 as an alternative dual imaging pair**. Figure 3 shows the 2PA spectra of EBFP2 (blue square) and mCherry (red circle) [3] grouped together with the EBFP2 (blue line) and mCherry (red line) fluorescence emission spectra. At 760 nm EBFP2 and mCherry have 2PA cross sections of 11 GM and 65 GM respectively, and the fluorescence emission maxima are separated by 165 nm.

Freed from the constraint of having to find FP pairs with large differences in Stokes shifts it is now possible to use FPs with higher two-photon cross sections and two-photon brightness. The higher excited state revealed in the tagRFP spectrum [3] makes it possible to couple this bright two-photon probe with mKalama1 such that a single wavelength can excite both optimally. The blue emission of mKalama1 is a limitation to this approach because of potential scattering and absorption in deep tissue imaging applications. This limitation, however, is balanced by the advantages of two photon excitation and access to multicolor imaging with only one laser. Further, the method of exploiting the different excitable transitions of FPs for simultaneous imaging is highly extensible, and one can easily imagine finding FP pairs with favorable properties such as further red shifted excitations and emissions, (Figure 3), greater photostability, and higher two-photon brightness.

## Methods

### Definitions

1. Energy transitions: the mitigators of absorption and fluorescence in FPs are the π electrons present in the chromophore. Upon excitation these electrons move into an excited, or higher energy state. The movement from the initial or ground state (S_0_) to the excited state (S_1 _or S_n_) is called an electronic transition whose frequency is proportional to the energy difference between the two states. Higher-energy transitions (S_0_→S_n_) occur with absorption at shorter (more energetic) wavelengths and lower-energy transitions (S_0_→S_1_) occur with absorption at longer (less energetic) wavelengths.

2. Cross section (σ_2_), GM, and two-photon brightness: The cross section (σ_2_) for two-photon absorption measured in GM (1 GM = 10^-50 ^cm^4 ^s) is the probability in which the fluorophore can simultaneously absorb a pair of photons per second at the unit average intensity of the incident light (1 photon/s * cm^2^) [16]. To obtain two-photon brightness (σ_2_×ϕ), the cross section (σ_2_) is multiplied by the fluorophore's quantum yield of fluorescence (ϕ).

3. Stokes shift: difference between excitation maximum and fluorescence emission maximum.

4. Kasha's rule states that fluorescence is independent of the mode or wavelength of excitation and thus exciting different transitions of a molecule will yield the same fluorescence emission spectrum. When exciting a higher-energy (shorter wavelength) transition, fluorescence does not occur from that state. Instead some of the energy is lost in the form of heat and emission occurs from the same lower-energy state as is the case when the lowest-energy state is excited directly [16].

5. Vibronic transitions: In addition to a change of electronic states upon optical excitation, a vibrational state of a molecule can also change. This implies that while in the ground (electronic and vibrational) state atoms are mostly occupying their near-equilibrium positions. In a vibrational excited state these atoms start to oscillate (acquire more kinetic energy) along certain normal coordinates of a molecule. If both electronic and vibrational states change upon excitation, this transition corresponds to the molecule acquiring a quantum of vibrational energy in the electronically excited state that sum to form a vibronic transition. This transition always occurs higher in energy (at shorter wavelength) than the pure electronic transition in the absorption spectrum [16].

### Protein expression and purification

The DNA encoding mKalama1, mAmetrine, the EBFP series, G1, G3, and the mTFP series were positioned in the pBAD plasmid (Invitrogen) such that a fusion protein is produced with an N-terminal 6xHis tag. Transformed E.coli (Top10, Invitrogen) were grown overnight in 4 mL LB + ampicillin. 200 mL of 0.2% arabinose, LB + ampicillin media was then inoculated with 1 mL of the overnight culture and allowed to grow at 34°C for 20 hours. The variants ECFP and mCerulean were positioned in the pRSET plasmid. For expression and purification protocol see [3]. Cells were lysed (Bugbuster, Novagen) and affinity purified with Ni-NTA His Bind Resin (Novagen). The proteins were eluted in imidazol buffer pH 8.

### Fluorescence lifetime, fluorescence quantum yield, extinction coefficient and concentration measurements

These methods have been previously described in detail in [3]. Briefly, fluorescence quantum yields for ECFP, mCerulean, G1, G3, mWasabi, and the mTFP series were measured relative to fluorescein. The fluorescence quantum yields for mKalama1, mAmetrine, and the EBFP series were measured relative to 9,10-Diphenylanthracene in cyclohexane. Extinction coefficients per chromophore and concentrations of mature chromophore were measured using the Strickler-Berg relation as described in [3]. See Table 2 for the data relevant to determining extinction coefficient using Strickler-Berg method.

### Two-photon absorption spectra and cross section measurements

Absolute 2PA cross sections were measured using relative fluorescence technique with coumarin 485 in methanol used as a standard for ECFP, mCerulean, mAmetrine, mKalama, and the EBFP series and fluorescein as a standard for G1, G3, mWasabi, and the mTFP series. For a description of the methods see [3].

Finding the optimal fluorescent proteins for TPLSM depends upon knowing the detailed structure of the 2PA spectra over a wide range of excitation wavelengths. Here we are continuing our work to use a common set of standards, and an all-optical approach of determining chromophore concentration, to quantitatively compare the cross sections of a broad series of FPs [3]. This approach is important because previous data obtained in different laboratories have varied [1,3,4,17,18].

### Imaging

To test the two-photon dual labeling approach with an imaging application HEK 293 cells were transiently transfected using lipofectamine 2000 (Invitrogen), with either mKalama1 or tagRFP in a CMV expression plasmid. The cells were subsequently mixed and re-plated so that they could be imaged simultaneously with one excitation wavelength (780 nm) and detected with two different PMTs (band pass filtered at 425-500 nm for mKalama1 and 550-575 nm for tagRFP). Excitation at 780 nm is most likely suboptimal, but this was as close to 760 nm that the Ti:sapphire laser used in the confocal microscope could be tuned to. To analyze the cross talk between the blue and red channels, the intensities for both the red and blue signals were plotted pixel by pixel.

## Authors' contributions

ST drafted the paper, developed new approach for dual imaging, expressed and purified the proteins, performed the photophysical characterization of the FPs, and imaged the cells. TEH helped draft the paper, helped develop the new dual imaging approach, and imaged the cells. NSM performed the photophysical characterization of the FPs. AR developed the experimental method for two-photon absorption measurements. MD drafted the paper, helped develop new approach for dual imaging and developed the methods of photophysical characterization of FPs. All authors read and approved the manuscript.
